# Disentangling emotional source memory: a mega-analysis on the effects of emotion on item-context binding in episodic long-term memory

**DOI:** 10.3389/fpsyg.2024.1459617

**Published:** 2024-12-30

**Authors:** Carlos Ventura-Bort, Yuta Katsumi, Janine Wirkner, Julia Wendt, Lars Schwabe, Alfons O. Hamm, Florin Dolcos, Mathias Weymar

**Affiliations:** ^1^Department of Biological Psychology and Affective Science, Faculty of Human Sciences, University of Potsdam, Potsdam, Germany; ^2^Department of Neurology, Massachusetts General Hospital and Harvard Medical School, Boston, MA, United States; ^3^Department of Clinical Psychology and Psychotherapy, Institute of Psychology, University of Greifswald, Greifswald, Germany; ^4^Department of Cognitive Psychology, Institute of Psychology, Universität Hamburg, Hamburg, Germany; ^5^Department of Psychology, Neuroscience Program, Beckman Institute for Advanced Science and Technology, University of Illinois Urbana-Champaign, Urbana, IL, United States; ^6^Faculty of Health Sciences Brandenburg, University of Potsdam, Potsdam, Germany

**Keywords:** emotion, episodic memory, long-term memory, source memory, mega-analysis

## Abstract

**Introduction:**

It has long been known that highly arousing emotional single items are better recollected than low arousing neutral items. Despite the robustness of this memory advantage, emotional arousing events may not always promote the retrieval of source details (i.e., source memory) or associated neutral information.

**Methods:**

To shed more light on these effects, we pooled data from seven different studies (*N* = 333) to investigate the role of emotion on item-context binding in episodic memory, as well as potential interacting factors (e.g., encoding instructions, type of retrieval task, or acute stress). In all studies, participants incidentally encoded common neutral objects (i.e., items), overlaid on different pleasant, neutral, or unpleasant background scenes (i.e., contexts). One week later, the encoded objects were presented intermixed with new ones and memory for item and source contextual details was tested, also considering the contribution of recollection and familiarity-based processes.

**Results:**

Linear mixed models revealed a recollection-based retrieval advantage for unpleasant and pleasant source contextual details compared to neutral ones. Bayes hypothesis-testing analysis further indicated decisive evidence in favor of a relevant role of emotional arousal and recollection in source contextual memory. Regarding item memory, linear mixed models revealed enhanced recollection-based memory for items encoded in pleasant contexts compared to their neutral and unpleasant counterparts. However, Bayes analysis revealed strong to moderate evidence for models without affective category (or its interactions), indicating that the affective category of contexts in which objects were paired during encoding had little influence on item memory performance.

**Discussion:**

The present results are discussed in relation to existing evidence and current neurobiological models of emotional episodic memory by also emphasizing the role of predictive processing as a useful conceptual framework to understand the effects of emotion on memory for source details and associated neutral information.

## Introduction

Whereas, most everyday experiences remain in our memory for only a few moments, highly arousing (i.e., pleasant and unpleasant) events such as the first bike ride, a wedding, or an injuring car accident may accompany us forever, as if they “almost left a scar in our cerebral tissue” (cf. James, 1890). Empirical evidence has well established the memory advantage for highly arousing single items (Bradley et al., 1992; Dolcos et al., 2005, 2017, 2020; Schiller et al., 2023; Weymar et al., 2009, 2011; Weymar and Hamm, 2013; Williams et al., 2022). This affective enhancement of memory is less associated with a familiarity-based (i.e., retrieval of an event without specifics) and more with a recollection-based experience (i.e., an elaborate process that includes the retrieval of specific details of the encoding event; Dolcos et al., 2005; Weymar and Hamm, 2013). In real-world situations, however, single items are rarely encountered in isolation, but are rather temporally dynamic (Palombo and Cocquyt, 2020; Bogdan et al., 2023), encoded with other information (Ranganath, 2010), and embedded in a particular context (Chiu et al., 2013). One question, thus, arises as to whether emotion also enhances the retrieval of details that are part of the emotional event (e.g., the *how, when*, or *where*, and associated neutral information)—i.e., *source* memory (Chiu et al., 2013; Dolcos et al., 2017; Mather and Sutherland, 2011; Mather et al., 2016; Murray and Kensinger, 2013; Squire et al., 2007). Available evidence suggests that despite the robustness of the memory enhancing effects of emotion on single items, affectively-laden information may sometimes *enhance* (D'Argembeau and Van der Linden, 2005; Doerksen and Shimamura, 2001; Graciela et al., 2016; Guillet and Arndt, 2009; Henson et al., 2016; Luck et al., 2014; Maratos and Rugg, 2001; Mather et al., 2009; Mather and Knight, 2008; Minor and Herzmann, 2019; Nashiro and Mather, 2011; Rimmele et al., 2012; Smith et al., 2004, 2005; Ventura-Bort et al., 2016a,b) or *impair* (Bisby et al., 2015; Bisby and Burgess, 2014; Cook et al., 2007; Ferré et al., 2019; Guo et al., 2018; Kensinger et al., 2007; MacKenzie et al., 2015; Madan et al., 2017, 2012; Mao et al., 2015; Mather et al., 2006, 2009; Mather and Knight, 2008; Nashiro and Mather, 2011; Rimmele et al., 2011; Touryan et al., 2007) the retrieval of source details (e.g., colors, spatial location, contexts, and temporal order of presentation) and associated neutral material (e.g., objects, words). These heterogeneous findings related to the effects of emotion on item-context binding in episodic source memory may be due to various factors (Mather et al., 2016; Bogdan et al., 2024), of which we consider the following two as critical: (1) the *prioritization* that the to-be-retrieved information receives and (2) the *retention interval* between encoding and retrieval.

According to the Arousal-Biased Competition (ABC) Theory (Mather and Sutherland, 2011), the modulatory effects of emotion on memory details depend upon the attentional priority that the critical information receives during encoding (e.g., via bottom-up perceptual salience, top-down attentional focus, or prior experience with particular stimuli). Emotion can enhance memory for source details or associated neutral items if they are highly prioritized, integrated in, or unitized with affective information, and can impair it if less prioritized or perceived as a competitor for resources against affective information (Mather et al., 2016; Mather and Sutherland, 2011). Neurobiological mechanisms for these opposing effects have been proposed in the *glutamate amplifies noradrenergic effects* (GANE) model (Mather et al., 2016). The GANE model suggests that prioritization of information occurs as a result of positive (or negative) feedback between glutamatergic neurons and noradrenergic varicosities of the locus coeruleus (LC) that potentiates (or diminishes) the neuronal activation associated with the mental representation of the prioritized (or non-prioritized) information. Importantly, the LC has widespread projections to brain regions that are thought to modulate encoding and consolidation of emotionally relevant information, including the hippocampus (e.g., Harley, 2007; Mello-Carpes and Izquierdo, 2013) and the amygdala (e.g., Chen and Sara, 2007; Clayton and Williams, 2000; McIntyre et al., 2012; Ventura-Bort et al., 2021; Williams et al., 1998), which are tightly linked to affective episodic memory (Dolcos et al., 2004, 2005, 2017; LaBar and Cabeza, 2006). It has, thus, been proposed that the enhancing effects of prioritization on source details or neutral items encoded with highly arousing information is supported by a positive interaction between glutamate and noradrenaline (NA) in these memory-sensitive regions (Mather et al., 2016).

In parallel to the fast, central phasic noradrenergic release that occurs after the encoding of affectively relevant information, slower physiological responses are initiated, resulting in the release of adrenal stress hormones (epinephrine and glucocorticoids). This peripheral hormonal release modulates central NA and corticosteroids levels in the amygdala and hippocampus (Henckens et al., 2009; McGaugh, 2000, 2004; McIntyre et al., 2012; Schwabe, 2017; Schwabe et al., 2022; Strange and Dolan, 2004), exerting special influence on memory consolidation processes (McGaugh, 2004; McIntyre et al., 2012; Schwabe et al., 2013; Strange and Dolan, 2004). Indeed, when memory for emotional and neutral items is tested after short retention intervals (e.g., 3–5 min after encoding)—diminishing the influence of peripheral catecholamines and glucocorticoids release in modulating central activity—, the recognition advantage for highly arousing vs. neutral items is less pronounced (Sharot et al., 2004; Sharot and Yonelinas, 2008; Schümann et al., 2017) compared to when tested after longer retention intervals (>24 h; Dolcos et al., 2005; Ritchey et al., 2008; Schümann et al., 2017; Segal and Cahill, 2009; Sharot et al., 2004; Sharot and Yonelinas, 2008; Weymar et al., 2009, 2010). These effects that may be mediated by enhanced consolidation processes may further extend to source memory retrieval as a memory advantage for source details paired with highly arousing (particularly unpleasant) compared with non-arousing information, has been found after long, but not short, retention intervals (Pierce and Kensinger, 2011).

Under the assumptions that high prioritization and long retention intervals may boost the impact of emotion on source memory and associated neutral items, we investigated—across a series of studies—their effect on emotional item-context binding on behavioral and neural level (Ventura-Bort et al., 2016a,b, 2020b, 2024 see also Table 1 for unpublished studies). In the first session, participants incidentally encoded common neutral objects (i.e., items), overlaid on different pleasant, neutral, or unpleasant background scenes (i.e., contexts). Later, the encoded objects were presented intermixed with new ones and memory for items and source contextual details were assessed.^1^ Critically, high prioritization was promoted by presenting the neutral objects prior to the scenes to ensure that they capture attentional resources. In addition, to facilitate the item-context binding, objects were not visually separated from the background scenes (Jaeger et al., 2009; Jaeger and Rugg, 2012; Smith et al., 2004, 2005), but directly overlaid on top of them. In addition, most studies included an encoding instruction to imagine the object as a part of the scene further facilitate item-context binding (Ventura-Bort et al., 2016a). Furthermore, we used a long retention interval (i.e., 1 week) to promote consolidation processes. To differentiate between familiarity and recollection processes we further used the Remember/Know procedure (Tulving, 1985) in most of the studies. In this task, participants indicate whether they can retrieve rich contextual details of the contiguous attributes of the encoding episode (i.e., Remember judgments), a process associated with recollection, or whether their retrieval lacks contextual specifics (i.e., Know judgments), a phenomenon associated with familiarity. Across studies, we observed consistent recollection-driven source memory enhancement for highly arousing source contextual information, but the expected memory advantage for items integrated in pleasant and unpleasant contexts was less reliable (e.g., Ventura-Bort et al., 2020b, 2024). In some cases, the memory advantage for objects from both pleasant and unpleasant contexts was observed (Ventura-Bort et al., 2020a), in others, these effects were exclusively found for items encoded in pleasant contexts (Ventura-Bort et al., 2016a,b), and in some studies, no differences were found in memory performance between items encoded in arousing and neutral contexts (Ventura-Bort et al., 2020b, 2019b; Wirkner et al., 2015; Buchwald et al., 2022). These findings indicate that even when high prioritization and long retention intervals are promoted, other factors may need to be considered to clarify the role of emotion in the retrieval of associated neutral information (Madan et al., 2017, 2019, 2020; Ventura-Bort et al., 2020a,b). Some such factors may be related to methodological differences between studies (see also Bogdan et al., 2024), which, in the current case, included variations in the encoding instructions, material used, lab environment, retrieval task employed, or the application of a standardized stress protocol.

**Table 1:** Summary of the study characteristics.

**References**	**Study**	**Gender W/M**	**Mean age**	**Protocol**	**Groups**	**Shortenedname**	***N***	**Encodinginstruction**	**Scene categories**	**Meanvalence**	**Meanarousal**	**Environment**	**Retrievaltask**	**Sourcelocation**	***N* new items**	***N* old items/ category**
Ventura-Bort et al. (2016b)	Study 1	26/3	22.7	–	Control	S1	29	Binding	P/U/N	6.94/2.73/5.27	5.84/5.93/3.51	EEG	O/N	Yes	144	48
Ventura-Bort et al. (2020a)	Study 2	26/4	21.8	–	Control	S2	30	Free viewing	P/U/N	7.02/2.58/5.08	5.88/6.00/3.37	EEG	R/K	Yes	180	60
Ventura-Bort et al. (2019b)	Study 3			Stress	Control	S3-C	30	Binding	P/U/N	7.02/2.58/5.08	5.88/6.00/3.37	EEG	R/K	No	90	60
		58/43	25.57		Stress	S3-S	39	Binding	P/U/N	7.02/2.58/5.08	5.88/6.00/3.37	EEG	R/K	No	90	60
					Stress Delay	S3-SD	18	Binding	P/U/N	7.02/2.58/5.08	5.88/6.00/3.37	EEG	R/K	No	90	60
Wirkner et al. (2015)	Study 4			Stress	Control	S4-C	24	Binding	U/N	2.73/5.27	5.93/3.51	EEG	O/N	Yes	96	48
		56/0	23.48		Stress	S4-S	32	Binding	U/N	2.73/5.27	5.93/3.51	EEG	O/N	Yes	96	48
Ventura-Bort et al. (2020b)	Study 5	26/3	26.68	–	Control	S5	29	Binding	P/U/N	7.14/2.34/5.13	6/6.06/3.25	MRI	R/K	No	132	44
Ventura-Bort et al. (2024)	Study 6	38/37	22.84	Stress	Control	S6-C	32	Binding	P/U/N	7.14/2.34/5.13	6/6.06/3.25	MRI	R/K	No	66	44
					Stress	S6-S	40	Binding	P/U/N	7.14/2.34/5.13	6/6.06/3.25	MRI	R/K	No	66	44
Buchwald et al. (2022)	Study 7	27/3	21.45	–	Control	S7	30	Binding	P/U/N	7.14/2.34/5.13	6/6.06/3.25	Eye tracker	R/K	No	132	44

Column information: Protocol indicates whether a standardized stress protocol was applied; Group indicates whether participants underwent an acute stress induction or not (i.e., control). Groups from studies without stress protocol were also coded as control; Shortened name contains the abbreviation of each study and group. N indicates the number of participants per group; Encoding instruction differentiates between active object/scene binding or passive viewing instructions; Scene categories were either Pleasant (P), Unpleasant (U), or Neutral (N). Mean Valence and Arousal refer to the mean normative score of the scenes used for each category. Environment codes the lab environment in which the experiment took place: EEG lab, eye tracker lab, or MRI scanner. Retrieval task indicates whether a Remember/Know (R/K) or Old/New (O/N) procedure was used. Source location indicates whether participants were instructed to also retrieve the location in which the object was presented during encoding. N New Items and N Old Items/Context codes the number of new items during retrieval and old items per category presented during encoding, respectively.

One useful approach in investigating this issue is data pooling, which provides an opportunity to comprehensively characterize the effects of emotion on long-term source memory. By increasing overall sample size, the pooling of independent studies with relatively homogeneous experimental designs may favor the exploration of interacting effects and the generalizability of the findings (Boedhoe et al., 2019; Giraudier et al., 2022). Therefore, we conducted a mega-analysis of seven studies (*N* = 333; methodological details of each individual study are summarized in Table 1), to clarify the impact of emotion and potentially interacting factors (i.e., encoding instructions, retrieval task, environmental settings, and stress protocol) on long-term memory performance for emotionally arousing contexts and associated neutral items.

Given that source memory and memory for associated neutral items embedded in emotional contexts may benefit from high prioritization and long-term retention periods (as promoted in the study designs), evidence for a better recollection-based, source contextual memory performance for both pleasant and unpleasant contexts compared to neutral contexts is expected. Similarly, evidence for recollection-driven enhancing effects for items encoded in highly arousing contexts is also hypothesized.

## Methods

### Participants

We pooled data from previously published and unpublished studies that investigated the effects of emotion on item/context binding in episodic memory. The total sample consisted of 333 healthy young adults (M_age_ = 23.5; female = 260, male = 73; Table 1 for gender distribution across samples). All participants provided informed written consent for the experimental protocol, which was approved in accordance with the declaration of Helsinki (Ethic's Approval number: MW 032014_rev_1). A summary of the studies is provided in Table 1.

### Stimulus material

In all studies, neutral objects were used as items and pleasant, unpleasant, or neutral pictures as contextual background scenes. Objects were extracted from two different datasets, *The Bank of Standardized Stimuli* (BOSS; Brodeur et al., 2012, 2014) and *the Ecological Adaptation of Snodgrass and Vanderwart* set (Moreno-Martínez and Montoro, 2012). Objects belonged to a variety of different semantic categories (e.g., office supplies, electronics, and household objects) and were grouped in six different sets carefully matched in terms of semantic category, familiarity, object agreement, and manipulability, according to the normative ratings of the standard samples (see BOSS and ecological adaptation of Snodgrass and Vanderwart norms). Background scenes were chosen from the *International Affective Picture System* (IAPS; Lang et al., 2008). Normative valence and arousal ratings were used to categorize the images as pleasant, unpleasant, and neutral. Each contextual background scene was paired with one item. To ensure that all items were equally paired with the different contextual background categories, item/context category pairings were counterbalanced across participants by creating different lists (for details of list construction see Ventura-Bort et al., 2019a, 2020b), in which items were assigned to different experimental conditions across lists (as old items paired with pleasant, neutral, or unpleasant contexts or as new items; see for details, Ventura-Bort et al., 2020b). Each participant was randomly assigned to one of the lists.

### Encoding task

**Figure 1:**
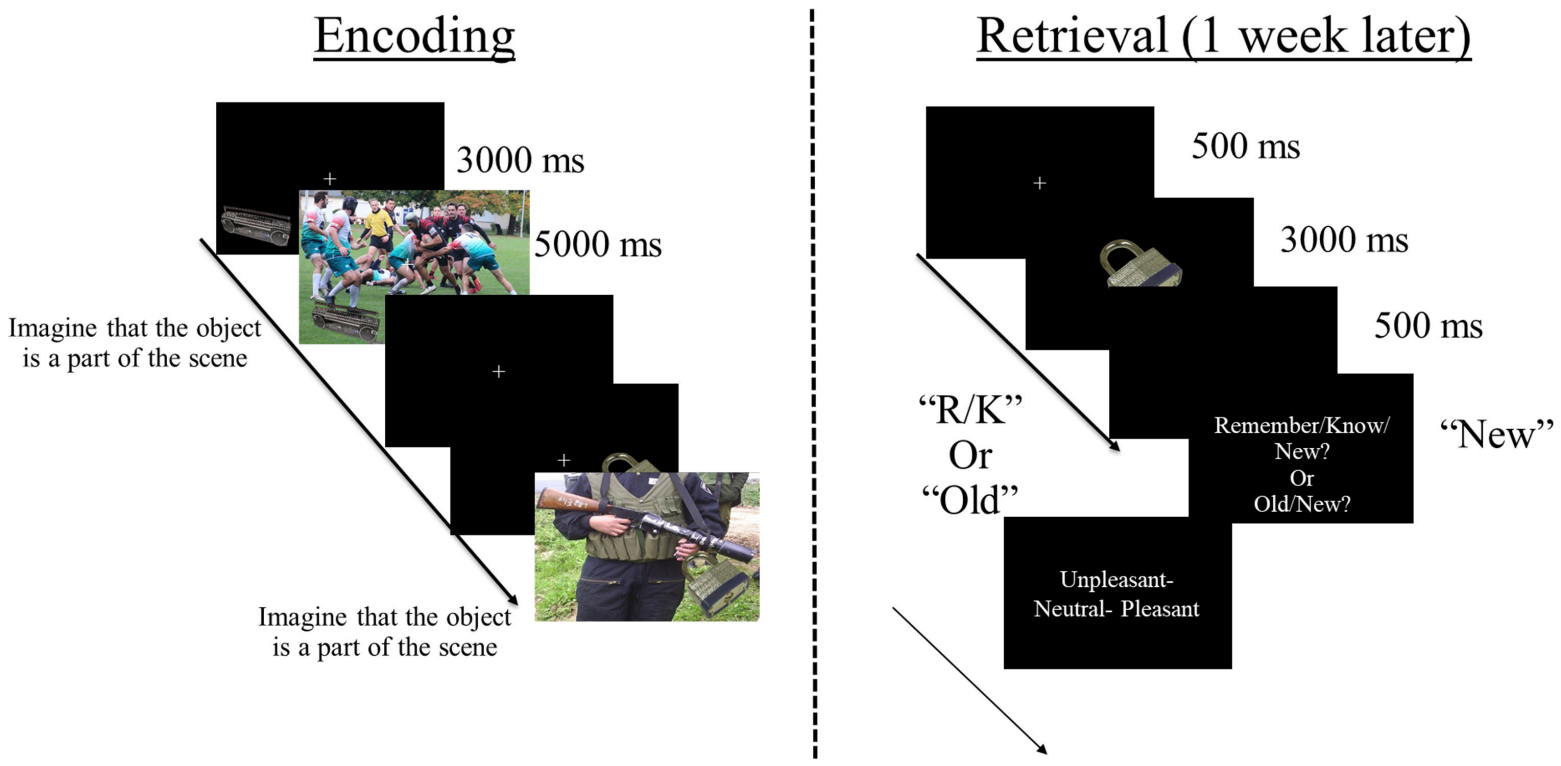
Schematic representation of the task design. During encoding, participants incidentally viewed different, everyday objects (office supplies, electronics, and household objects) presented in one of the four quadrants of the screen, overlaid on different background scenes that were either pleasant, neutral or unpleasant. In most studies (1, 3–7; except study 2) participants were also instructed to imagine that the object is a part of the scene during viewing the object/scene combination. During retrieval, participants viewed the encoded objects intermixed with new objects and performed a recognition task, using either a Remember/Know paradigm or an Old/New paradigm. If objects were recognized as old [i.e., Remember (R), Know (K), or Old judgments], participants were asked to retrieve the associated scene background (pleasant, unpleasant, or neutral).

During encoding, items were superimposed on contextual background scenes (Figure 1). The number of item/context pairings encoded varied across studies (see Table 1). Each trial began with the presentation of an item in one of the four quadrants of a black screen. After 3,000 ms, a pleasant, unpleasant, or neutral contextual scene was added as background. The presentation of the item/context pairing lasted 5,000 ms. During that time—in studies 1, 3–7—participants were instructed to imagine the object as a part of the scene—to facilitate item-context binding—and to indicate whether the imagination was successful or not by pressing a button after item/context offset (e.g., Ventura-Bort et al., 2016a,b). In study 2, the item/context pairings were presented in a free viewing condition without an active binding instruction (Ventura-Bort et al., 2019a, 2020a). In all studies, a fixation cross was presented continuously during all item/context trials, to which participants were instructed to look at during the task. Participants were never informed about the subsequent retrieval task (i.e., incidental encoding).

### Retrieval task

Approximately 1 week after encoding, participants came back to the lab to perform the retrieval task in which previously encoded items were presented intermixed with new ones in a pseudo-randomized fashion (Figure 1). The number of new items varied across studies (see Table 1). In each recognition trial, an item was presented in the center of the screen without context for 3,000 ms. After item offset, a question was shown to which participants were instructed to indicate whether the item was seen during encoding. In studies 2, 3, 5–7, the Remember/Know procedure was implemented (Tulving, 1985) and the “Remember/Know/New?” question was asked here. For that, participants were instructed to press the “Remember” button when they recognized the item as shown during encoding and could bring back specific associated information that occurred during encoding (e.g., thoughts evoked by the object when seen for the first time). The “Know” button was required when the item was recognized as presented during encoding but without retrieval of specific associated information, and participants were instructed to press the “New” button when the item was not seen during encoding.^2^ In studies 1 and 4, participants were presented with the “Old/New?” question and instructed to press the “Old” button if they recognized the item as old or else the “New” button.

If participants made “Old” judgments (“Remember” or “Know” judgments in studies 2, 3, 5–7), follow-up questions about source information were presented. In all studies, participants were instructed to indicate the category (e.g., pleasant, unpleasant, and neutral) of the contextual background scene that was paired with the item during encoding. In studies 1, 2, and 4, prior to the context-related question, participants were asked to retrieve the location the item was presented at during encoding (i.e., which quadrant of the screen). In all studies, tasks were programmed with Presentation (Neurobehavioral Systems Inc., Albany, CA, USA).

### Lab environment

The included studies took place in three different lab environments. Studies 1–4 were carried out in an EEG lab. These experiments took place in a sound-attenuated, dimly lit room and participants were seated in a comfortable chair (e.g., Ventura-Bort et al., 2016a,b). Participants in studies 5 and 6 performed both the encoding and retrieval session in the MRI scanner (Ventura-Bort et al., 2016a,b). Study 7 took place in an eye-tracking lab. Here, the participants' head was positioned in an eye tracker and eye movements were continuously recorded during encoding and retrieval.

### Stress protocol

In studies 3, 4, and 6, participants underwent a stress protocol prior to the encoding session. In each of the three studies, participants were randomly assigned to either a stress or control condition. In the stress condition, participants were exposed to the Socially Evaluated Cold Pressure Test (SECPT; for stress and control protocol, see Schwabe et al., 2008; Schwabe and Schächinger, 2018) followed by a difficult mental arithmetic test (see for similar test, Smeets et al., 2012). The stress induction lasted 15 min. After, participants were informed about the two-part protocol by a cold and unsociable experimenter, in the first part, they were asked to immerse their right hand down to the wrist into ice water (temperature: 0–3°C) for 3 min (or until they could no longer tolerate it). During hand immersion, participants were instructed to look straight into a camera because their faces were videotaped. They were told that video recordings would later be analyzed for facial expressions. Thereafter participants performed the second part of the stress condition protocol consisting of a difficult 5-min mental arithmetic test in which they had to count backwards as fast and accurately as possible in steps of 17 starting at 2043. Whenever they counted too slowly or mistakenly, they received negative feedback (i.e., to count faster or start over again at 2043).

In the control condition, participants were received by a friendly and socially interacting experimenter. Firstly, participants immersed their right hand down to the wrist for 3 min in warm water (35–37°C). They were not videotaped. Secondly, they performed a simple arithmetic task in which they had to count consecutively from 1 to 25 at their own pace and had to start anew at 1 when having reached 25. In all three studies, participants in the control and stress condition performed the encoding session ~30 min after the stress induction (control) protocol. A subgroup in study 2 (stress delay), performed the encoding session 180 min after the stress induction.

### Behavioral data recording and analysis

Behavioral performance for items and source contextual information was recorded using Presentation (Neurobehavioral Systems Inc., Albany, CA, USA) and analyzed using RStudio. Data preprocessing was conducted using *tidyverse* (Wickham et al., 2019). Memory performance for items (i.e., objects) and source contextual details (i.e., emotional category of background scenes) was examined in two different sets of analysis. D prime [d'; *z*(P Old) – *z*(P False Alarm)] was used as an index for item memory. For source contextual memory, which was assessed based on participants' responses to the emotional category of the contextual background scene that was paired with the object during encoding, the unbiased hit rate (Hu) was calculated (Ventura-Bort et al., 2020a; Wagner, 1993). The *Hu* index takes into account not only the stimulus performance but also the judge performance and is defined as the conjoint probability of the correct identification of a stimulus and the correct use of a response (Wagner, 1993; see for details Ventura-Bort et al., 2020b).

In the first set of analyses, memory performance was coded independently of memory type. Therefore, Remember and Know judgments that were obtained in studies using the Remember/Know procedure, were indistinctively coded as “Old” judgments.

In the second set of analyses, a distinction between Remember and Know judgments was made. It is important to note that analysis of familiarity and recollection processes in explicit memory retrieval is constrained by the assumption that both processes are interrelated. However, this relation can be exclusive or independent (Yonelinas and Jacoby, 1995). Theoretical proposals and empirical data clearly support the independence assumption as a more appropriate index to differentiate between recollection and familiarity processes (Yonelinas and Jacoby, 1995; Yonelinas, 2002). Thus, we decided to control for dependency (see for details Ventura-Bort et al., 2020a). d' was therefore calculated under the independence assumption for both Remember (d′ Recollection = *z*(P Remember) – *z*(P False Alarm Remember)) and Familiarity:


$$\begin{array}{l} \text{z}\left(\frac{P\;Know\;Hit\;Rate}{1-P\;Remember\;Hit\;Rate}\right)-z\left(\frac{P\;Know\;False\;Alarm}{1-P\;Remember\;False\;Alarm}\right) \end{array}$$

For source memory, the interaction effects of context and memory processes were analyzed by calculating the Hu indexes for each affective category and memory judgment, separately. Specifically, the Hu indexes for Remember and Know judgments, were calculated by only taking into consideration items that were judged as remembered or known, respectively. For instance, for neutral contexts of objects restricted to Know judgments, the Hu was calculated as follows:


$$\begin{array}{l} \frac{Hit\;Know\;Neutral\;Context}{\left(Hit\;Know\;Neutral\;Context+Incorrect\;Know\;Neutral\;Context\right)}\\ {}*\frac{Hit\;Know\;Neutral\;Context}{N^{{}^{\circ}}\;of\;times\;Neutral\;Context\;is\;chosen\;under\;Know\;judgements}\; \end{array}$$

*Hit Know Neutral Context* = Number of objects paired with neutral contexts retrieved based on Know judgments and whose background category was correctly identified*; Incorrect Know Neutral Context* = Number of objects paired with neutral contexts retrieved based on Know judgments and whose background category was not correctly identified; *N*° *of times Neutral Context is chosen under Know judgments* = Number of objects whose background was labeled as “Neutral,” including those whose background was wrongly identified.

For each participant, memory performance was averaged across affective category (e.g., pleasant, neutral, and unpleasant) and, when required, split by memory type (i.e., Remember, Know).

The effects of affective category on memory for items (as indexed by d') and source contextual details (as measured by Hu) were tested with linear mixed models (LMM) using *lme4* (Bates et al., 2012). In the first set of analysis, as fixed effects, we specified *Affective category* (e.g., pleasant, neutral, and unpleasant), *Encoding instructions* (i.e., binding, free viewing), *Group* (i.e., control, stress, and stress delay), *Retrieval task* (Old/New, Remember/Know procedure), *N new items* (i.e., number of new items presented during retrieval), and *Environment* (i.e., EEG, MRI, and Eye tracker) and their associated interactions. In the second set of analyses, the fixed factor *Memory type* (i.e., Remember, Know) was further added. *Participant* as well as a new dummy variable called *Study group* consisting of a combination of *Group* (i.e., control, stress, and stress delay) and *Study* (i.e., studies 1–7) were modeled as random effects.

Because the main focus of this mega-analysis was to clarify the role of potential factors modulating memory performance, particularly in interaction with *Affective category*, the effects of methodology-related factors were included in the analyses (i.e., *Encoding instructions, Retrieval task, N new items, Environment*, and *Group*) using a parsimonious model selection [following the general recommendations by Bates et al. (2012) and without knowledge or consideration of fixed-effect estimates]. Notably, the parsimonious model selection always considered the fixed-effect estimates of *Affective category*. For model comparisons, the χ^2^-distributed likelihood ratio and its associated *p*-value was used. All analyses were conducted using full information maximum likelihood modeling. If significant effects were found (or exploratory analysis conducted), they were followed up by *post-hoc* comparisons using *lsmeans* (Lenth, 2016), correcting for multiple comparisons (Tukey's honest significant difference; HSD; Tukey, 1949).

Although the significant effects (*p* < 0.05) that could arise from the mixed model analyses may inform about the probability, under the assumption of no difference between conditions (H_0_; i.e., no effects of affective category on memory performance are observed), of obtaining a result equal to or more extreme that what was actually found, they do not inform about the extent of acceptance/rejection of the alternative hypothesis (H_1_; i.e., positive effects of affective category on memory performance) *per se* (Greenland et al., 2016; Held and Ott, 2018). To address this issue, we additionally used Bayes analysis (Wagenmakers et al., 2018) to evaluate our hypotheses. Using a Bayesian approach for hypothesis testing encompasses the calculation of the predictive adequacy of two competing models, to quantify the evidence provided by the data for one model over the other (Wagenmakers et al., 2018). To test the evidence in favor of the alternative hypotheses, we calculated the Bayes factor (BF_10_) on the significant effects by comparing final models to null models (i.e., models without the significant effects of interest). For instance, to test the effects of *Affective category* on item memory, the model including such a factor will be tested against an identical model without the *Affective category* factor. To interpret the results of the Bayes factors, the following classification was used (Lee and Wagenmakers, 2013): a BF_10_ larger than 100 provides decisive evidence in favor of H_1_, a value between 30 and 100, indicates very strong evidence for H_1_, a score between 10 and 30 provides moderate evidence for H_1_, a value between 1 and 3 indicates anecdotal evidence for H_1_, a BF_10_ of 1 provides no evidence for either H_1_ or H_0_. On the other hand, values between 0.3 and 1 provide anecdotal evidence for H_0_, values between 0.1 and 0.3 indicate moderate evidence for the H_0_, scores between 0.03 and 0.1 show strong evidence for H_0_, values between 0.01 and 0.03 indicate very strong evidence for H_0_, and values lower than 0.01 provide decisive evidence for H_0_.

## Results

Tables 2–5 contain the results of the linear mixed models for all analyses.

**Table 2:** Linear mixed model predicting memory performance for items independently of memory type (*N* participants = 333; *N* observations = 943).

	**D prime**				
**Predictors**	***b***	***SD***	***CI***	***t***	***p***
(Intercept)	0.54	0.09	0.37 – 0.72	6.04	**< 0.001**
***Affective category***					
**Pleasant**	**0.06**	**0.02**	**0.02 – 0.09**	**3.18**	**0.002**
Unpleasant	0.01	0.02	−0.02 – 0.04	0.55	0.582
***Encoding instructions***					
**Binding**	**0.62**	**0.10**	**0.42 – 0.82**	**5.98**	**< 0.001**
***Retrieval task***					
**Old/new**	**0.90**	**0.07**	**0.75 – 1.04**	**12.17**	**< 0.001**
***Lab***					
**MRI**	**0.31**	**0.07**	**0.17 – 0.45**	**4.37**	**< 0.001**
Eye tracker	0.13	0.10	−0.07 – 0.33	1.32	0.189

Bold values indicate significant effects, *p* < .005.

### Memory for items

#### Effects of affective category independently of memory type

The most parsimonious model that described the data best included—in addition to *Affective category—*the factors *Encoding instructions, Retrieval task*, and *Environment* as fixed factors (Table 2). Interactions between fixed factors did not improve the model and were thus not included.

**Figure 2:**
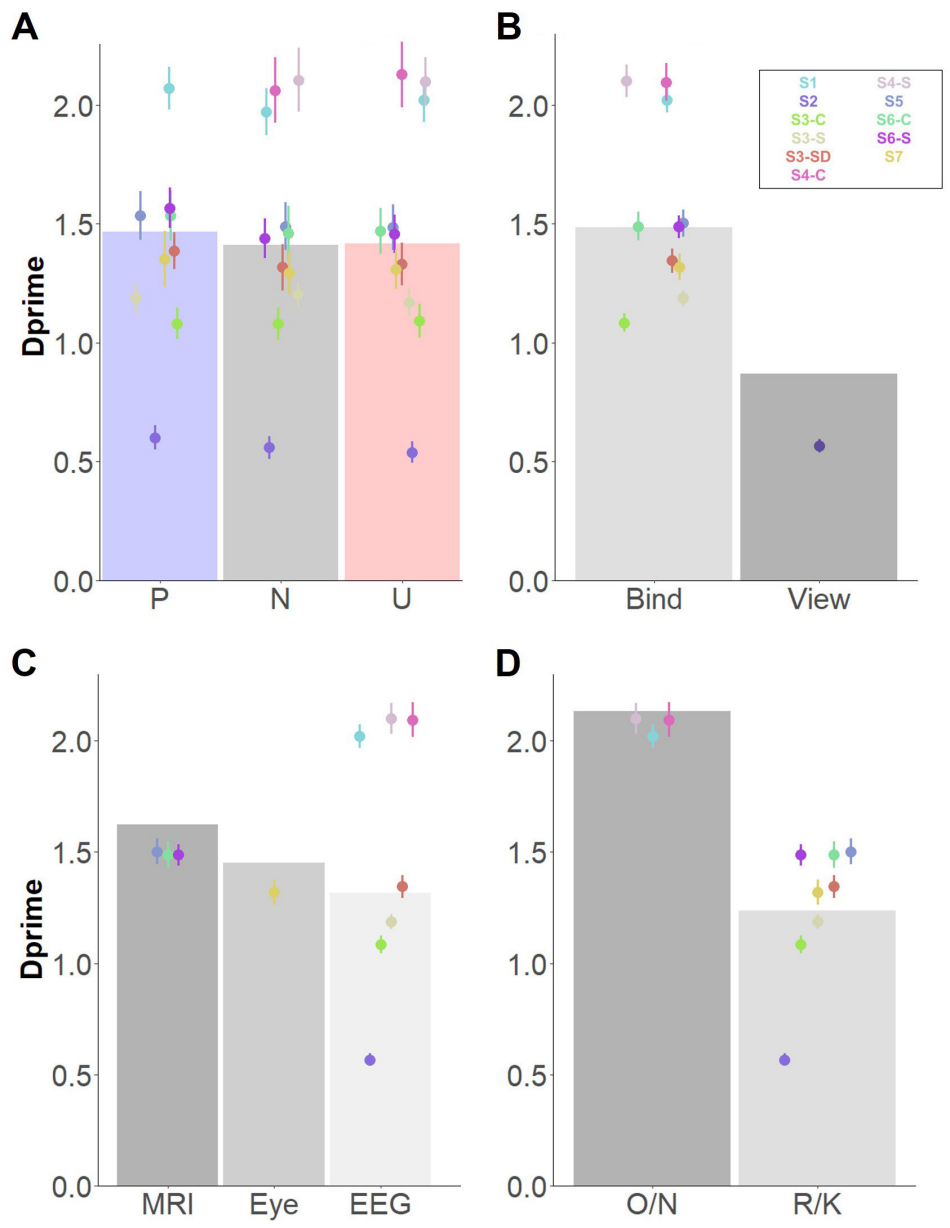
Main significant effects for memory for items, independently of memory type. Dots depict averaged values across study and group. Error bars represent standard errors. Bar plots depict the estimated means from the linear mixed model. **(A)** Main effect of affective category; **(B)** Main effect of encoding instructions; **(C)** Main effect of retrieval task; **(D)** Main effect of Environment. O/N, Old/New task; R/K, Remember/Know procedure.

Results showed an effect of *Affective Category*, with higher memory performance for items from pleasant (but not from unpleasant) compared with neutral contextual background scenes (Figure 2A). Follow-up analysis revealed higher memory performance for objects encoded in pleasant, compared to both neutral, *t*_(610)_ = 3.18, *p* = 0.004, and unpleasant contexts, *t*_(610)_ = 2.66, *p* = 0.02, but no memory differences were found between objects from unpleasant and neutral backgrounds, *t*_(608)_ = 0.55, *p* = 0.84. However, the Bayes factor indicated strong evidence in favor of a model without the *Affective Category* factor (BF_10_ = 0.24; see Table 6).

A significant effect of *Encoding instructions* revealed better memory performance when participants were instructed to bind objects and background scenes, compared to when they were asked to attentively view the item/context pairings (Figure 2B). An effect of *Retrieval task* was also found, indicating that memory performance was better when the Old/New task was used, in comparison to the Remember/Know procedure (Figure 2C). Finally, a significant effect of *Environment* revealed higher memory performance in the MRI scanner (but not in the Eye tracker environment), compared to the EEG lab (Figure 2D). *Post-hoc* comparison confirmed differences between the EEG and MRI environment, *t*_(5.04)_ = 4.29, *p* = 0.017, but no differences were observed between either the EEG and eye tracker, *t*_(5.87)_ = −1.31, *p* = 0.44, or MRI and eye tracker environments, *t*_(5.65)_ = 1.73, *p* = 0.27. The effects of *Encoding instructions, Retrieval task*, and *Environment* were supported by decisive evidence in favor of inclusion of these factors in the model (BF_10_s > 100).

#### Effects of affective category as a function of memory type

The simplest, best fitting model included—in addition to *Affective category–* the factors *Memory type, Encoding instructions*, and *Environment* as fixed factors. Including interactions between *Memory type* and *Affective category* improved the model significantly. No other fixed factors or interactions between fixed factors explained the data better and were thus not included (Table 3).

**Table 3:** Linear mixed model predicting item memory performance as a function of memory type (*N* participants = 247; *N* observations = 1,481).

	**D prime**				
**Predictors**	***b***	***SD***	***CI***	***t***	***p***
**(Intercept)**	**1.02**	**0.05**	**0.92 – 1.13**	**18.67**	**< 0.001**
***Memory type***					
**Remember**	**−0.21**	**0.05**	**−0.30** to **−0.12**	**−4.63**	**< 0.001**
***Affective category***					
Pleasant	0.02	0.05	−0.06 – 0.11	0.54	0.592
Unpleasant	0.00	0.05	−0.09 – 0.09	0.01	0.993
***Encoding instructions***					
**Binding**	**0.48**	**0.09**	**−0.66** to **−0.30**	**−5.19**	**< 0.001**
***Lab***					
**MRI**	**0.26**	**0.06**	**0.14 – 0.38**	**4.12**	**< 0.001**
**Eye tracker**	**0.18**	**0.09**	**0.00 – 0.36**	**1.98**	**0.049**
**Remember** ^*^Pleasant	**0.16**	**0.06**	**0.04 – 0.29**	**2.54**	**0.011**
Remember ^*^ Unpleasant	0.08	0.06	−0.04 – 0.21	1.27	0.205

Bold values indicate significant effects, *p* < .005.

**Figure 3:**
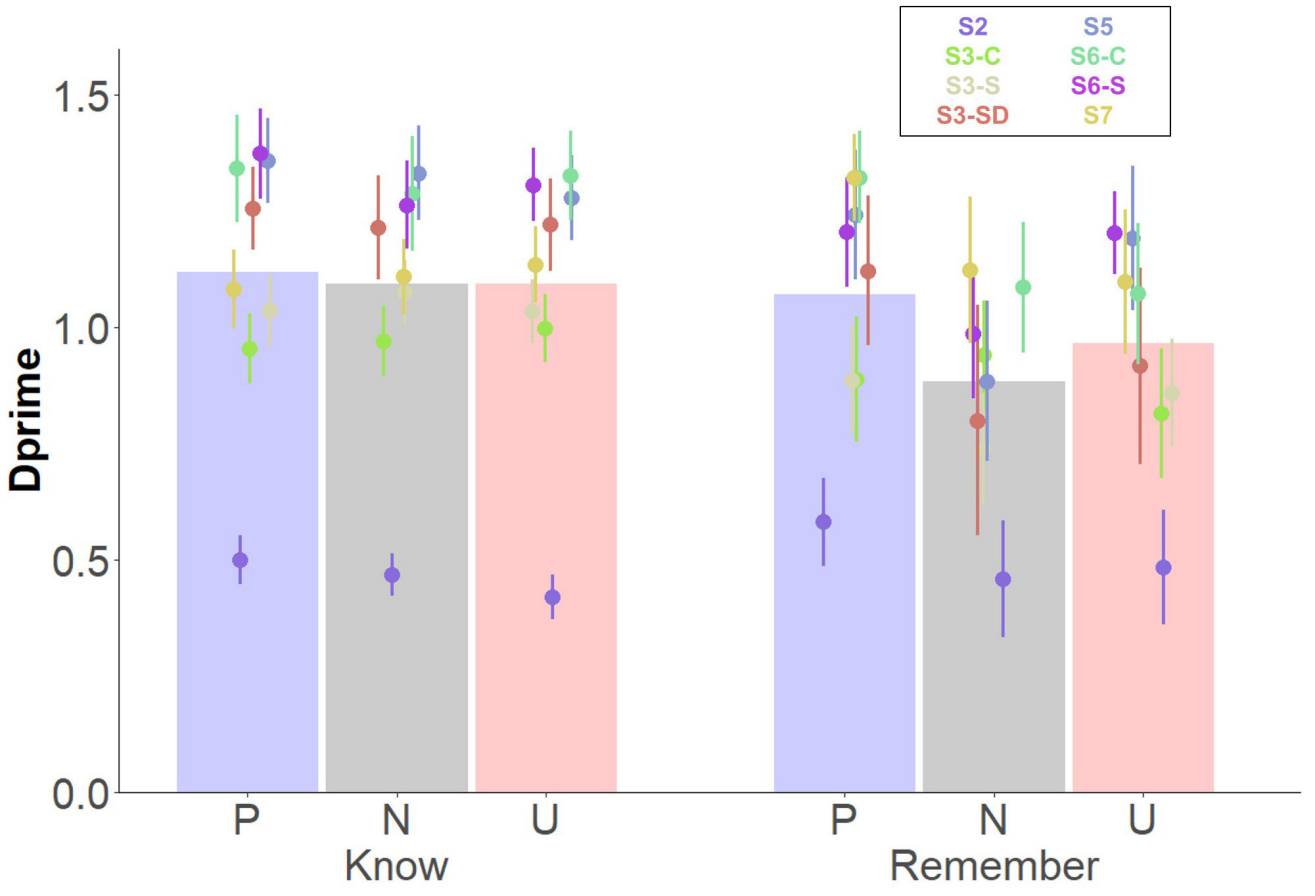
Interacting *Affective category* and *Memory type* effects on memory performance for items. Dots depict averaged values across study and group. Error bars represent standard errors. Bars depict the estimated means from the linear mixed model for the interaction *Affective category* * *Memory type*.

A *Memory type* effect revealed higher memory performance under Know than Remember judgments. As in our previous model, significant effects of *Encoding Instructions* and *Environment* were found (see also Table 6 for Bayes factors). Interestingly, in the absence of a significant main effect of *Affective Category*, a significant interaction effect between *Memory type* ^*^Affective category was found, indicating higher memory performance for objects embedded in pleasant backgrounds compared with neutral ones, particularly when memory was based on recollection (Remember judgments; Figure 3). These results were confirmed in follow-up analyses, indicating no differences between affective categories in Know judgments [pleasant vs. neutral: *t*_(1, 229)_ = 0.54, *p* = 0.85; pleasant vs. unpleasant: *t*_(1, 229)_ = 0.52, *p* = 0.86; unpleasant vs. neutral: *t*_(1, 229)_ = 0.01, *p* = 0.99], but higher memory performance for objects from pleasant contexts for Remember judgments [pleasant vs. neutral: *t*_(1, 229)_ = 4.13, *p* < 0.001; pleasant vs. unpleasant: *t*_(1, 229)_ = 2.33, *p* = 0.05]. However, no differences were found between objects from unpleasant and neutral contexts: *t*_(1, 229)_ = 1.80, *p* = 0.17. Despite the significant interacting effects, the Bayes Factor indicated moderate evidence in favor of a model without the interaction *Memory type* ^*^ *Affective Category* (BF_10_ = 0.1).

### Memory for source contextual details

#### Effects of affective category independently of memory type

**Figure 4:**
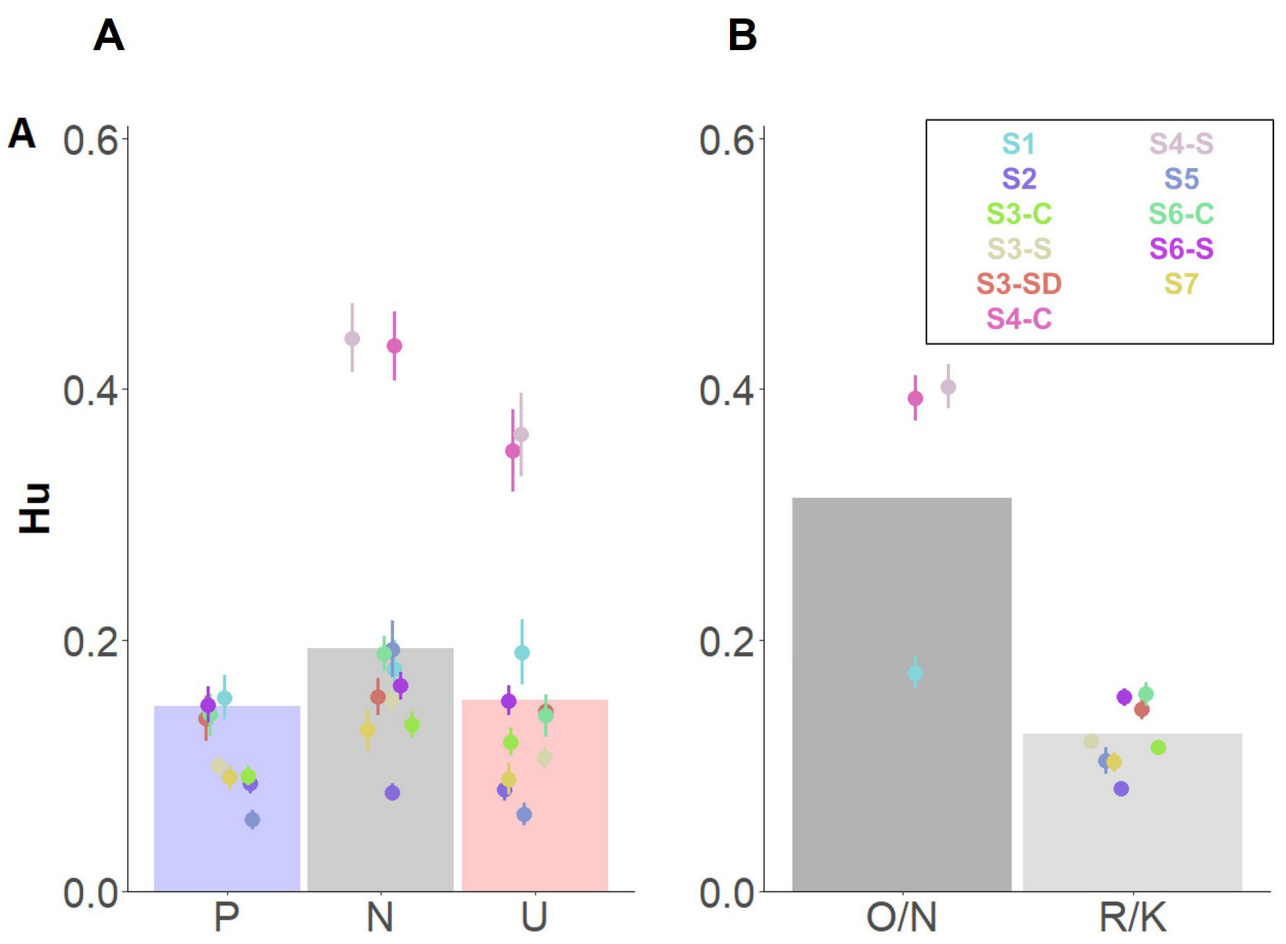
Main effects of source memory independently of memory type. Dots depict averaged values across study and group. Error bars represent standard errors. Bar plots depict the estimated means from the linear mixed model. **(A)** Main effect of *Affective category*; **(B)** Main effect of *Retrieval task*. O/N, Old/New task; R/K, Remember/Know procedure.

The simplest, best fitting model included –in addition to *Affective category–* the fixed factors *Encoding instruction*, and *Retrieval task*. Because interaction effects did not improve the model, they were not included. Results revealed a significant effect of *Affective category*, indicating higher memory performance for neutral, compared to pleasant and unpleasant contexts (Table 4, Figure 4A). However, no differences between pleasant and unpleasant contexts were observed, *t*_(608)_ = −0.79, *p* = 0.71. Moreover, *Retrieval task* effects revealed higher source memory performance when participants executed the Old/New task, in comparison with the Remember/Know procedure (Figure 4B). The Bayes factor indicated decisive evidence in favor of the inclusion of the three factors in the model (BF_10_s > 100).

**Table 4:** Linear mixed models predicting source memory performance independently of memory type (*N* participants = 333; *N* observations = 940).

	**Hu**				
**Predictors**	***b***	***SD***	***CI***	***t***	***p***
(Intercept)	0.11	0.07	−0.02 – 0.24	1.69	0.091
***Affective category***					
**Pleasant**	**−0.05**	**0.01**	**−0.06** to **−0.03**	**−7.24**	**< 0.001**
**Unpleasant**	**−0.04**	**0.01**	**−0.05** to **−0.03**	**−6.91**	**< 0.001**
***Encoding instructions***					
Binding	0.05	0.07	−0.09 – 0.18	0.66	0.506
***Retrieval task***					
**Old/new**	**0.19**	**0.05**	**0.10 – 0.28**	**4.17**	**< 0.001**

Bold values indicate significant effects, *p* < .005.

#### Effects of affective category as a function of memory type

When memory was split into Remember and Know judgments, the best fitting model included –in addition to *Affective category–* the fixed factors *Memory type, Environment*, and *Encoding instructions*. The interactions between *Memory type* and *Affective category*, between *Memory type* and *Environment*, and between *Memory type, Affective category*, and *Group* were also included, given that they improved the model fit (Table 5). A significant main effect of *Affective category* revealed overall higher memory performance for neutral contexts. A main effect of *Encoding instructions* was also observed, with better source memory performance when object/scene pairings were actively bound during encoding. The Bayes factors indicated decisive evidence in favor of the inclusion of these factors in the model (BF_10_s > 100; see Table 6). A significant interaction between *Memory type* ^*^ *Affective category* revealed higher memory performance for both pleasant and unpleasant contexts when memory was mediated by recollection (Remember judgments; BF_10_ > 100). Follow-up analyses confirmed higher memory performance for objects receiving Remember, compared to Know judgments, for pleasant, *t*_(1, 216)_ = 8.13, *p* < 0.001, and unpleasant contexts, *t*_(1, 215)_ = 6.07, *p* < 0.001, but not for neutral ones, *t*_(1, 215)_ = 0.31, *p* = 0.75. *Environment* also interacted with *Memory type*, showing higher source memory performance related to Remember judgments in the MRI scanner and Eye tracker lab compared to the EEG environment (BF_10_s > 100). *Post-hoc* comparisons showed higher contextual memory under Remember than Know judgments in the three environments, *t*s > 3.71, *p*s < 0.001, but recollection-based memory was higher in the MRI, compared to the EEG environment, *t*_(4.05)_ = 5.91, *p* = 0.009. No other differences were observed (|*t*s| < 2.15, *p*s > 0.205).

**Table 5:** Linear mixed model predicting source memory performance for source details as a function of memory type (*N* participants = 247; *N* observations = 1,479).

	**Hu**				
**Predictors**	***b***	***SD***	***CI***	***t***	***p***
**(Intercept)**	**0.14**	**0.02**	**0.11 – 0.18**	**8.54**	**< 0.001**
***Memory type***					
Remember	−0.03	0.02	−0.07 – 0.01	−1.56	0.12
***Affective category***					
**Pleasant**	**−0.05**	**0.02**	**−0.09** to **−0.02**	**−3.07**	**0.002**
**Unpleasant**	**−0.04**	**0.02**	**−0.07** to **−0.01**	**−2.26**	**0.024**
***Lab***					
MRI	0.01	0.01	−0.02 – 0.04	0.77	0.444
Eye tracker	−0.02	0.02	−0.06 – 0.03	−0.82	0.411
***Encoding instructions***					
**Passively watching**	**−0.04**	**0.02**	**−0.08 – 0.06**	**−2.11**	**0.035**
**Memory type** ^*^Affective category					
**Remember** ^*^ **Pleasant**	**0.14**	**0.02**	**0.09 – 0.19**	**5.84**	**< 0.001**
**Remember** ^*^ **Unpleasant**	**0.08**	**0.02**	**0.03 – 0.12**	**3.12**	**0.002**
**Memory type** ^*^Lab					
**Remember** ^*^ **MRI**	**0.08**	**0.02**	**0.04 – 0.11**	**4.52**	**< 0.001**
**Remember** ^*^ **Eye tracker**	**0.07**	**0.03**	**0.01 – 0.12**	**2.53**	**0.012**
**Memory type** ^*^ **Affective Category** ^*^ **Group**					
Know^*^Neutral^*^Stress	0.01	0.02	−0.04 – 0.05	0.30	0.763
Remember^*^Neutral^*^Stress	−0.01	0.02	−0.05 – 0.04	−0.26	0.793
Know^*^Pleasant^*^Stress	0.01	0.02	−0.05 – 0.03	−0.45	0.652
**Remember** ^*^ **Pleasant** ^*^ **Stress**	**0.05**	**0.02**	**0.01 – 0.10**	**2.20**	**0.028**
Know^*^Unpleasant^*^Stress	−0.02	0.02	−0.07 – 0.02	−1.00	0.318
**Remember** ^*^ **Unpleasant** ^*^ **Stress**	**0.09**	**0.02**	**0.04 – 0.13**	**3.87**	**< 0.001**
Know^*^Neutral^*^Stress Delay	0.02	0.04	−0.05 – 0.05	−0.81	0.418
Remember^*^Neutral^*^Stress Delay	−0.03	0.04	−0.11 – 0.05	−0.81	0.418
Know^*^Pleasant^*^Stress Delay	0.04	0.04	−0.04 – 0.12	0.89	0.376
Remember^*^Pleasant^*^Stress Delay	−0.00	0.04	−0.08 – 0.08	−0.01	0.995
Know^*^Pleasant^*^Stress Delay	0.03	0.04	−0.05 – 0.11	0.03	0.486
Remember^*^Pleasant^*^Stress delay	0.01	0.04	−0.07 – 0.09	0.34	0.736

Bold values indicate significant effects, *p* < .005.

**Table 6:** Summary of the main findings of the Bayes factor to test for the significant effects observed in the best fitting models for item memory and source contextual memory.

	**Model description**	**Formula**	**Bayes factor output (BF**_**10**_ **)**							
			**Affective category**	**Encoding instruction**	**Retrieval task**	**Lab**	**Memory type**	**Affective category** ^*^Memory type	**Memory type** ^*^Lab	**Affective category** ^*^ **Memory type** ^*^ **Group**
Memory for items	Effects of affective category independently of memory type	*D' ~ Affective category + Encoding instructions + Retrieval task + Lab*	0.024	>100	>100	>100	—	—	—	—
	Effects of affective category and Memory type	D' ~ Affective category ^*^ Memory type+ Encoding instructions + Lab	0.027	>100	—	>100	14.61	0.11	—	—
Memory for contexts	Effects of affective category independently of memory type	*Hu*~*Affective category + Encoding instructions + Retrieval task*	>100	>100	>100	—	—	—	—	—
	Effects of affective category and Memory type	*Hu*~Affective category ^*^ Memory type + Memory type ^*^ Environment + Encoding instructions + Affective category: Memory type: Group	>100	62.27	—	>100	>100	>100	>100	0.29

**Figure 5:**
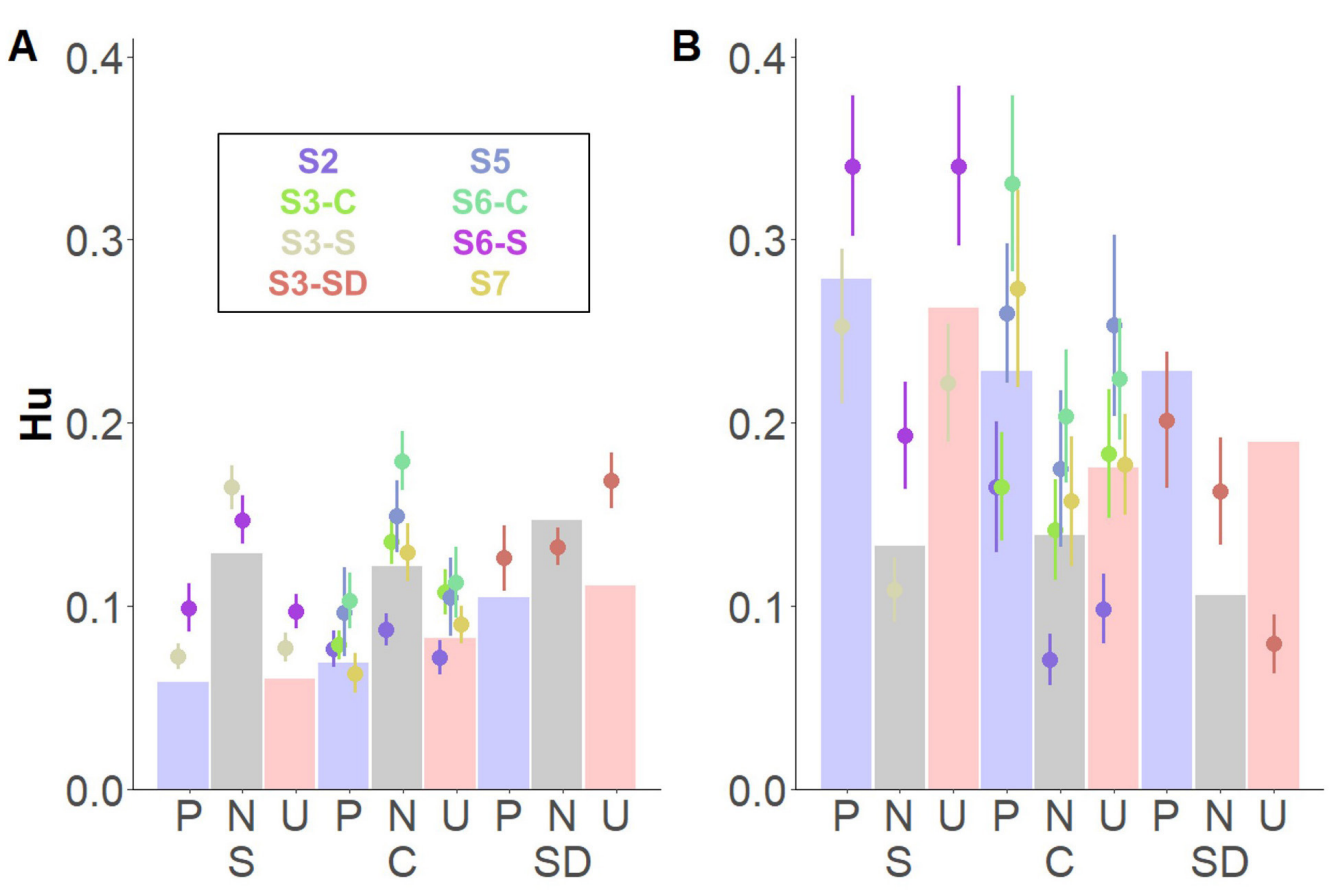
Interacting effects of *Affective category, Memory type*, and *Group* for memory for source details. Dots depict averaged values across study and group. Error bars represent standard errors. Bar plots depict the estimated means from the linear mixed model. **(A)** Depicts know judgments and **(B)** remember judgments both split by group: stress (S; left), control (C; middle), and stress delay (SD; right).

Finally, a three-way interaction between Memory type^*^ Affective Category^*^ *Group* revealed source memory performance for pleasant and unpleasant contexts based on Remember judgments, especially for participants in the stress group (Figure 5). Following-up on the interacting effects, we carried out *post-hoc* comparisons between groups on source memory for each memory type and affective category, separately. For familiarity-based contextual memory, no differences between groups on any of the affective categories were observed (*p*s > 0.99; Figure 5A). However, for recollection-based memory, a trend emerged, indicating that participants in the stress, compared to the control, showed higher memory performance for unpleasant contexts: *t*_(21.5)_ = 3.86, *p* = 0.057 (Figure 5B). No further significant differences were found (|t*s|* < 2.20, *p*s > 0.73). Bayes factor provided moderate evidence in favor of a model without the triple interaction effect (BF_10_ = 0.29).

## Discussion

The primary goal of the current study was to further our understanding of the effects of emotion on memory for source contextual details and associated neutral items. Capitalizing on a large sample size (*N* = 333) derived from pooling together data from seven different studies with similar experimental designs, we investigated the modulatory effects of emotion as well as potential interacting factors (e.g., encoding instructions, type of retrieval task, lab environment, or the application of a stress protocol) on memory binding for associated items (objects) and source contextual details (emotional category of background scenes). Linear mixed models revealed a recollection-based retrieval advantage (i.e., uniquely for Remember judgments) for unpleasant and pleasant source contextual details compared with neutral ones. Bayes hypothesis-testing analysis further indicated decisive evidence in favor of the interacting affective category (pleasant vs. unpleasant vs. neutral) and memory type (recollection vs. familiarity) effects, providing support for a relevant role of these factors in source contextual memory. Regarding item memory, linear mixed models uncovered enhanced memory for items encoded in pleasant contexts compared with their neutral and unpleasant counterparts, particularly for Remember judgments. However, Bayes analysis revealed strong to moderate evidence for models without *Affective Category* (or its interactions), indicating that the affective context in which objects were placed during encoding had little influence on item memory performance. Furthermore, we observed decisive evidence for modulating effects of encoding instructions, retrieval task, and lab environment. Below, we will discuss these results with regards to available evidence and the current neurobiological models of emotional episodic memory and also add *predictive processing* as a theoretical framework that may be useful in understanding the effects of emotion on memory for source details and associated neutral information.

Based on the existing neurobiological models of emotional episodic memory (e.g., McGaugh, 2004; Mather et al., 2016), the current studies (all part of the mega analysis) were designed to promote the positive effects of emotion on source memory, by facilitating item-context integration (i.e., high prioritization of items) and fostering consolidation processes. Under these circumstances, a recollection-based memory advantage for highly arousing (both pleasant and unpleasant) source contextual details was observed in our pooled data analysis. Contrary to our hypothesis, however, no evidence was found for effects of emotion on memory for associated neutral information (i.e., item memory). Neuroimaging studies have shown that the hippocampus, as well as connected cortical regions within the posterior parietal cortex (King et al., 2015; Ranganath and Ritchey, 2012; Rugg and Vilberg, 2013) may play an important role in recollection-based processes (Eichenbaum et al., 2007; Yonelinas et al., 2002), especially for highly arousing material (Maddock et al., 2001, 2003). Interpreted within the above-mentioned models (Mather et al., 2016; McGaugh, 2004), high prioritization and long retention period may have facilitated the initial encoding of emotionally relevant information (Kensinger, 2009; Salsano et al., 2024), by engaging limbic and para-limbic regions (e.g., Pedale et al., 2019) and the subsequent NA and corticosteroids action in memory sensitive regions, such as the hippocampus, favoring both encoding and consolidation of affectively-laden contextual details that led to better recollection-based retrieval.

Although no evidence in favor of additional interacting effects of affective category with other experimental manipulations was found, memory performance was modulated by encoding instructions, retrieval task, and environmental settings in isolation. Participants' memory performance was better when they were instructed to actively bind objects and background scenes relative to when they just attended to pairs of stimuli, individually. During the “binding” instructions, participants were asked to effortfully combine item/context pairings which likely triggered deeper encoding than during the relatively effortless “just viewing” instructions. These findings are in line with earlier studies showing that the depth of processing enhances subsequent memory retrieval (Craik and Tulving, 1975; Hanslmayr et al., 2009).

In addition to the instructions given during encoding, the task used during retrieval also modulated memory performance. Specifically, memory for items and contexts was better when using the simpler Old/New task compared to the Remember/Know procedure. Previous studies testing the effects of retrieval tasks on memory performance have consistently found differences between these two paradigms (Eldridge et al., 2002; Gardiner et al., 1998; Hicks and Marsh, 1999). The usage of a single Remember/Know procedure compared with a two-step procedure (i.e., the Old/New question followed by the Remember/Know question), has been associated with a more liberal response bias (Hicks and Marsh, 1999) and more false alarms, particularly for “Know” judgments (Eldridge et al., 2002), suggesting that memory judgments are susceptible to differences in task instructions. Our findings replicate these observations showing decreased discriminability of item and source information under the Remember/Know procedure.

We also found that memory performance was modulated by the environment in which the task was conducted. Memory for items was enhanced in the MRI compared to the EEG environment. In the same vein, higher recollection-based memory for contextual scenes was found in the MRI setting. One of the unique characteristics of the MRI environment in relation to other experimental settings is the loud scanner noise that is constantly delivered during the task. This noise, which is often perceived as annoying, aversive or stressful could influence cognitive processes while participants perform tasks in the scanner. Previous studies have shown that the scanner environment not only increases cortisol levels (Tessner et al., 2006) but such a moderately stressful event might also favor task engagement, leading to performance improvements (Plessow et al., 2011). To systematically test whether the influence of MRI noise on cognitive performance, Hommel et al. (2012) investigated whether task performance in an MRI environment was modulated by the presence vs. absence of MRI noise. The authors observed that scanner noise favors cognitive control by reducing the influence of potential distractors (Hommel et al., 2012). Our results may thus indicate that the MRI environment helped participants stay focused on the encoding and retrieval tasks, resulting in better memory performance, particularly compared to the EEG environment.

Altogether, our findings emerging from the mega-analysis of prior individual studies suggest that highly arousing contexts facilitate the encoding and subsequent retrieval of source contextual details. Existing neurobiological models of emotional memory postulate that under high prioritization and long retention intervals that promote consolidation processes (Mather et al., 2016), and as such, the memory enhancing effects of emotional events may extend to associated neutral information. However, inconsistent with the prior interpretation, for item memory we found strong evidence in favor of models without the *Affective Category* factor (or interactions), suggesting that alternative perspectives may also have to be considered to understand the relationship between emotion and memory source details and associated neutral information (see also Bogdan et al., 2024, for evidence regarding the impact of emotion on item-context binding when the emotional information is manipulated in the items' content).

An alternative view explaining our emotional source memory findings could be the *Predictive Processing account* (e.g., Clark, 2013; Friston et al., 2017; Hutchinson and Barrett, 2019; Hohwy, 2013), which posits that the brain is an active entity that is continuously making predictions about the future (e.g., Bastos et al., 2012; Friston, 2010; Rao and Ballard, 1999; Sterling and Laughlin, 2015). Relevant for the present study, predictive processing has been integrated into recent accounts of affective processing as well as its impact on learning and memory, and the associated neural mechanisms (Cross et al., 2018; Ferreira-Santos, 2016; Kalbe and Schwabe, 2020, 2022; Meaux et al., 2019; Rouhani et al., 2023; Strube et al., 2021).

Critically, the predictive processing framework can be used to further understand the current inconsistent memory findings for item and source contextual details. Because the brain is constantly generating predictions about incoming sensory inputs based on past experience and encoding prediction errors, brain activity and ensuing mental experience in a given trial occur as a function of what one has experienced in previous trials, suggesting that brain activity and affective experience observed over the course of an experiment are temporally dependent. Furthermore, predictions about sensory stimuli can develop over a longer timescale (e.g., across the lifespan), which inevitably vary from one subject to another and may be hard to modify through exposure to stimuli in a laboratory setting (Lee et al., 2021). One possibility, therefore, is that the processing and subsequent retrieval of contextual scenes is influenced by previously encountered events that determine the probability of their occurrence to a greater extent than that of isolated items (e.g., Strube et al., 2021). The recollection-based advantage for emotionally arousing source contextual details observed in the present data pooling study could thus be partly the consequence of their unpredictability (i.e., prediction error) in comparison to neutral contexts (Schwartz, 1997; Schwartz et al., 2002). On the other hand, the formation of item-context associations may be simultaneously influenced by different factors that have opposing effects on memory. Although emotional (unpredictable) contexts may favor the storage of the encountered information, the resources devoted to process the details of the composition (i.e., precision signals) could both enhance the memory of the associated items, if such resources are dedicated to item processing, or diminish it if invested in the processing of other details. The interplay of these opposing effects could have, thus, also led to the lack of evidence of emotional effects on item memory. Future studies may therefore also use a predictive processing framework to interpret the effects of emotion on memory for source details and associated neutral information and also considering sources of variability (e.g., chain of previously seen events, personal experiences with similar contexts; c.f., recent work on source memory: e.g., Ben-Yakov et al., 2022; Greve et al., 2017; Kafkas and Montaldi, 2018; Kalbe and Schwabe, 2020, 2022; Ortiz-Tudela et al., 2021; Quent et al., 2022; Van Kesteren et al., 2012).

In contrast to individual studies, the mega-analysis approach used here benefits from a larger sample size to draw more solid conclusions. However, it should be noted that the samples of the current studies were relatively homogeneous and included young healthy adults, mostly women. The homogeneity of the collapsed samples may pose constraints for the generalization of the results to older and more gender-balanced populations. This is particularly important considering that previous studies have reported gender (Canli et al., 2002; De Goede and Postma, 2008; Guillem and Mograss, 2005) and age differences in memory retrieval (Rhodes et al., 2019). Future studies investigating the impact of these demographic characteristics on emotional source memory would lead to further insights in this field. Additionally, future studies should also consider combining mega-analysis with other machine learning-based validation approaches like cross-validation leave-one-out approaches that could further inform about the reliability and generalizability of the results across samples.

## Conclusions

In the current study, we aimed at extending our understanding of the effects of emotion on item/context memory binding. Pooling data from seven different studies (*N* = 333), we observed a recollection-based emotional enhanced source contextual memory, in line with existing neurobiological models of emotional episodic memory (Mather et al., 2016; McGaugh, 2004). However, for item memory Bayes hypothesis-testing revealed strong evidence in favor of models without the *Affective Category* factor. The current findings also invites to consider alternative perspectives, such as the predictive processing, to better understand the relationship between affective relevance and source memory. Future work might benefit from considering sources of variability (e.g., chain of previously seen events, personal experiences with similar contexts) that are otherwise labeled as random error in clarifying when and why affective relevant information might show differential effects on source memory.

## Data Availability

The datasets presented in this study can be found in online repositories. The names of the repository/repositories and accession number(s) can be found at: https://osf.io/bxdvg/.

## Footnotes

^1^In source memory studies, item memoranda are usually cued, and participants must indicate whether they were previously seen or not during the encoding episode. Source memoranda refer to the recognition of associated information and is typically assessed only when items have been identified as old (e.g., Smith et al., 2004). Additionally, although source and relational memory have been interchangeably used (Eichenbaum et al., 2007) methodological differences between source and relational memory studies have been pointed out (e.g., Chiu et al., 2013). Source memory studies typically examine how, when or where a stimulus was presented (that is the source). These studies use a limited number of alternative choices for each modality (e.g., for when: first or second block; for where: upper or lower part of the screen). On the other hand, relational memory studies assess a more association between items and details of the encoding episode that implies a one-to-one relationship. That is, relational information is trial-unique and as numerous as trials presented (Chiu et al., 2013; Bogdan et al., 2024). Acknowledging these methodological differentiations, here, we refer to source memory when talking about memory for contextual categories and to item memory when describing memory for objects.

^2^As part of the instructions, participants were also given the following example: Imagine that you are in the supermarket, and in front of you there is a person with a red t-shirt that is talking to her partner about their holidays. Few days later, you see the same person on the street. If you can then recognize the person and retrieve any contextual information about the first encounter (where you saw her for the first time, what she was wearing, what she was talking about, what you thought when you saw her), that is an example of “remembering” her. However, if you can recognize her, but are not able to identify from where, or retrieve any specifics, then is an example of “knowing” her.
