# Supplementary material for: Disentangling emotional source memory: a mega-analysis on the effects of emotion on item-context binding in episodic long-term memory
Source: Front Psychol. 2024 Dec 30;15:1459617. doi: 10.3389/fpsyg.2024.1459617 (PMC11727367; doi:10.3389/fpsyg.2024.1459617)
Supplement: Supplementary file 1 [file Data_Sheet_1.docx]

Appendix 1.

List of objects used in experiments:

BOSS: armclock01, xcutter03a, cket01a, lculator01, ain01, opper01, oppydisk02a, yingpan02a, irdryer02a, at03b, ighheelshoe01, ighlighter02b, ourglass, ey01, eaf02a, ighter01, atch, itten04, apertowel, ictureframe04, illow01a, lunger02, earviewmirror, emotecontrol04, ing01, ollingpin01a, ewingmachine01a, poon01, portbag, praybottle01, tapleremover, trainer02, tudiolight, hreeholepunch03, oyanimal02, iolin, ateringcan, ackpack01a, attery02b, eermug01a, ottlecap, halkboarderaser01, hessboard, hisel02b, lipboard, omputermouse06, ottonpad, iaper01c, envelope03a, ilmroll, lashlight02b, loortile01, iftbag01a, lasses01a, andbag02a, andfan01b, andmixer01d, eadphones02b, orsefigurine, ifejacket, fficechair01, ainttray, inecone01, late01b, eflector, idinghelmet, afetypin, cale01a, cissors01, crubbingbrush05b, ippycup, ponge01, praycan, wimgoggles, ambourine01, eabag, hermometer02b, issuebox01b, oothbrush03b, shirt, atch02a, inerack, andaid01, ib, leachbottle, oot02b, ane, arkey, hristmasball, igarette, ompactpowder, ookiecutter, orkscrew03a, raser, xpandablefolder03a, xpansioncard, lobe, anger02a, osenozzle, interlockingbuildingblock03b, kitchenscale01a, laptop01a, lipstick02a, lockingplier, medal02b, notebook03a, pen04b, petcarrier, plasticbasket01a, powercable, printer02, propanetank01, puzzlepiece, rope03, scarf, screwdriver04b, starfish01, suitcase, teapot, adjustablewrench01b, baseball01a, bracelet01, broom01, camera01a, candle08b, cclamp, divider02, electricrazor, flask, glass02a, glassmop, hairclip03, icepack, iceskate, kettle01, lock03a, microscope, milkcontainer, mousetrap, nailpolish03b, pingpongpaddle01a, pitcher02b, scotchtape, squeegee01b, staple, taperecorder, thimble, thread02, thumbtack02a, umbrella04, usbkey, vase02, wallet02a, wheel01, whisk, antenna, telephone01b, number3, balloon01b, birdie, bowl01, bungeecord02, cardboardbox, chalkboard01, cooler01, dice05a, drill01b, dustpan, fan, fishinghook03, foodprocessor, fork03c, iron01b, jar02, keychain, lbracket01, magnifyingglass01b, makeupbrush04, monitor, nailclipper03b, outletadapter01, paintroller01, peeler01, pencilsharpener02a, plant01, playingcard, pliers02b, pot02a, radio01, sock01a, speaker04, sunglasses04, tennisball01b, toaster01, toiletpaper01a, tripod01, tweezers02a, vikingmask01b, woodboard, strawbasket01, bag, belt02a, bikehelmet, binder03b, boosterseat, bubbleblower, cassettetape01a, cd, cellphone, clothespin03b, coffeepot03a, comb02a, computerkeyboard02, drum01, earring01, exercisebench, fireextinguisher01, flyswatter, gluestick, hinge, icecubetray01a, log, lunchbox, mallet01b, manshoe, nail, pacifier02d, paperclip01b, peppermill02b, perfume01a, pylon01, razor01, saw02b, stapler03a, tongs01b, videocamera01a, walkman, waterbottle01b, 8ball, beerbottle, bench01, bikepump01, blender, candelabra, djmixer02, drumset, football, gamecontroller01, harmonica, lamppost01, lectern01, megaphone, poloshirt, radiator, soccerball, telescope, tennisracket, acousticguitar02, alarmsystem, anchor, barrel01, basketball01, bicycle, callbell, carjack, chest01, grandpiano, horseshoe, hubcap, ladder, mailbox02, rubikcube, accordion01, airconditioner, crosscountryski, doorhandle, dresser02, drinkshaker, dvdcase01, fishingrod, funnel, kite, pin, sheriffhat, skigoggles01, weedwacker, wheelbarrow01, cornet, armchair02, awning, bikelock, birdnest, boxingglove01, carsidemirror01, cymbal, dartboard, drumstick, golfbag, jeans01, masquerademask01, pokerchips, record, scooter, stool01, tie02, watervalve, witchhat, amphora, apron, banjo, baseballglove, bed, bowrake, clarinet, clover, diploma02, doghouse, ironingboard01, trampoline, wallclock, woodenshoe, dreamcatcher, balalaika, bathtub, beachumbrella01, canoepaddle02, cookingpot, discoball, dolly01, electricguitar01, filingcabinet, foosballtable, frisbee, graduationcap, gravyboat, jumpercables, punchingbag, zipper.

The ecological adaptation of Snodgrass and Vanderwart: Bookcase, Skate, Skirt, Trowel, Bathrobe, Couch, Diabolo, Fondue, Harp, Undershirt, Wardrobe, Jacket, Rocking chair, Skittle, Ludo, Saxophone, Table, chair_M, Bedside_table, Coat, Showel, Sofa.

List of images used in experiments: 1019, 1090, 1113, 1205, 1220, 1304, 1440, 1463, 1540, 1590, 1595, 1620, 1640, 1650, 1659, 1660, 1710, 1720, 1721, 1722, 1811, 1930, 1932, 2026, 2038, 2045, 2075, 2102, 2104, 2155, 2160, 2190, 2191, 2206, 2208, 2210, 2211, 2214, 2272, 2273, 2300, 2345, 2347, 2359, 2372, 2377, 2383, 2390, 2393, 2396, 2400, 2410, 2411, 2500, 2506, 2512, 2513, 2593, 2595, 2840, 2850, 2870, 2980, 3015, 3016, 3019, 3051, 3059, 3064, 3100, 3102, 3110, 3140, 3150, 3180, 3181, 3190, 3191, 3195, 3213, 3225, 3280, 3530, 4598, 4599, 4611, 4643, 4645, 4653, 4658, 4659, 4660, 4668, 4680, 4687, 4690, 4694, 4695, 4698, 4800, 5390, 5470, 5471, 5510, 5520, 5535, 5621, 5623, 5626, 6212, 6241, 6243, 6370, 6410, 6520, 6560, 6562, 6563, 6571, 7033, 7037, 7041, 7061, 7130, 7140, 7161, 7170, 7179, 7192, 7205, 7234, 7493, 7495, 7500, 7504, 7546, 7547, 7550, 7590, 7595, 7710, 7920, 8001, 8021, 8041, 8080, 8158, 8170, 8180, 8185, 8186, 8191, 8300, 8370, 8470, 8480, 8490, 8620, 9042, 9210, 9230, 9250, 9253, 9301, 9360, 9414, 9420, 9425, 9490, 9520, 9560, 9561, 9570, 9599, 9600, 9622, 9635.1, 9902, 9904, 9908, 9910, 9921, 9927, 9930
